# Obstructive sleep apnea and lung cancer: molecular underpinnings and clinical translational prospects

**DOI:** 10.3389/fcell.2026.1764594

**Published:** 2026-02-13

**Authors:** Lin Zhang, Fengling Liu, Junyao Li

**Affiliations:** Department of Respiratory and Critical Care Medicine, The Second Hospital of Jilin University, Changchun, China

**Keywords:** chronic intermittent hypoxia (CIH), lung cancer, obstructive sleep apnea (OSA), sleep fragmentation (SF), tumor microenvironment (TME)

## Abstract

Obstructive sleep apnea (OSA) is one of the most common sleep-disordered breathing conditions, characterized by repetitive narrowing or collapse of the pharyngeal airway, associated with chronic intermittent hypoxia (CIH), sleep fragmentation (SF), and sympathetic hyperactivity. Recent epidemiological surveys have shown that OSA may be associated with adverse outcomes, including various diseases and even death. In particular, its association with lung cancer has attracted widespread attention: on the one hand, OSA may promote tumor progression and reduce treatment sensitivity via core mechanisms such as chronic inflammation and oxidative stress; on the other hand, lung cancer itself and its related therapies can conversely exacerbate OSA, forming a complex bidirectional interplay that remains to be fully elucidated. This narrative review systematically searched PubMed and Web of Science databases for literature on OSA and lung cancer published up to September 2025, with a specific focus on mechanistic and clinical observational studies. It aims to clarify the inherent links between the pathophysiological features of OSA and the lung cancer tumor microenvironment (e.g., exosomes, tumor-associated macrophage polarization, and cancer stem cells), further shedding light on the underlying molecular mechanisms, and deepening the understanding of the pathogenic pathways driving OSA-associated lung cancer initiation and progression. Ultimately, this study aims to provide new insights into the clinical management of this comorbid condition and holds significant implications for improving the prognosis of patients with this condition.

## 1 Introduction

Obstructive sleep apnea (OSA) and lung cancer pose enduring public health challenges and exacerbate the medical pressures and economic burdens around the world. OSA is a common sleep-disordered breathing condition characterized by recurrent collapse and obstruction of the upper airway during sleep, resulting in intermittent hypoxemia and accompanied by daytime sleepiness (An et al., 2025). The major pathophysiologic characteristics of OSA are currently regarded as comprising CIH, SF, and sympathetic hyperactivity. Studies have shown that CIH and SF lead to increased oxidative stress and chronic inflammation, immune dysregulation, and imbalances in body homeostasis, ultimately driving carcinogenesis (Javaheri and Javaheri, 2020; Cao et al., 2015; Yuan et al., 2024). Moreover, OSA has been associated with other diseases, such as chronic respiratory disease (McNicholas, 2017; Locke et al., 2022), cardiovascular disease (Drummond et al., 2003; Yeghiazarians et al., 2021), cognitive dysfunction (Felmet and Petersen, 2006), diabetes (Abelleira et al., 2024), and metabolic disorders (Almendros et al., 2022). An updated estimate based on publicly available data worldwide in 2019 indicated that nearly one billion people have OSA, with prevalence exceeding 50% in some countries (Benjafield et al., 2019). Such widespread population impact underscores the public health significance of investigating the association between OSA and lung cancer.

Lung cancer remains one of the most common malignant tumors globally and the leading cause of cancer-related deaths. While the incidence and mortality of lung cancer have shown a downward trend in countries such as the United Kingdom and the United States, driven by the popularization of early detection technologies and advances in targeted therapy, immunotherapy, and other advanced treatment modalities (Siegel et al., 2025; Shelton et al., 2024), the global landscape of lung cancer prevention and control remains grim. In 2022, an estimated 20.0 million new cancer cases and 9.7 million cancer deaths were reported worldwide, of which lung cancer accounted for 2.5 million new cases (12.4%) and 1.8 million deaths (18.7%) (Bray et al., 2024). Consequently, identifying novel risk factors for lung cancer—especially those reversible through clinical intervention—may play a crucial role in its prevention and treatment.

In recent years, the association between OSA and lung cancer has emerged as a cross-disciplinary research hotspot. A meta-analysis of observational research revealed that OSA correlates with increased lung cancer risk in studies with at least 5 years of median follow-up (Cheong et al., 2022). In addition, studies have also shown a high prevalence of OSA among patients with lung cancer (Cabezas et al., 2019; Bhaisare et al., 2022). Furthermore, clinical studies have confirmed that OSA may be associated with increased lung cancer mortality, an association that is more prominent in patients aged <65 years old (Nieto et al., 2012). Additionally, a large multicenter French Cohort suggested an association between nocturnal hypoxemia and lung cancer (Justeau et al., 2020), which was further confirmed *in vivo* and *in vitro* experiments (Kang et al., 2020). Notably, data from intervention studies suggest that OSA may be a modifiable risk factor for cancer. In a 5-year prospective observational study, Gómez-Olivas et al. found that treatment with continuous positive airway pressure (CPAP) improves the prognosis of melanoma compared with untreated moderate to severe OSA (Gómez-Olivas et al., 2023). And Khalyfa et al. discovered that plasma-derived exosomes from patients with Obesity hypoventilation syndrome (OHS) promote lung cancer cell growth, and long-term CPAP treatment (3, 12, and 24 months) can gradually reverse this effect (Khalyfa et al., 2022). However, existing research remains controversial—studies by Gozal et al. and Sillah et al. failed to find an association between OSA and increased lung cancer risk or cancer-related mortality (Sillah et al., 2018; Gozal et al., 2016). This controversy highlights the necessity of in-depth analysis of the molecular mechanisms underlying the interaction between OSA and lung cancer.

Although numerous studies have explored the association between OSA and lung cancer, comprehensive studies integrating molecular mechanisms, clinical findings, and therapeutic strategies remain relatively scarce. Current mechanistic studies mostly focus on isolated pathways (e.g., HIF-1α, βARs signaling), ignoring the differential molecular effects and synergistic interactions of CIH and SF, as well as failing to establish a unified regulatory network centered on the tumor microenvironment (TME). Additionally, the regulatory roles of lung cancer heterogeneity in the interaction mechanism have not been fully elucidated, which not only limits the clinical applicability of mechanistic conclusions but also hinders the development of subtype-specific therapeutic strategies.

Accordingly, this narrative review focuses on “molecular mechanism integration and therapeutic potential translation.” By systematically searching the PubMed and Web of Science databases (up to September 2025), we included mechanistic research (cellular/animal experiments, human tissue sample detection), clinical observational studies, systematic reviews/meta-analyses, and narrative reviews. This review focuses on three key aspects: first, systematically dissecting the differential molecular pathways through which core OSA pathophysiological features (CIH, SF, sympathetic hyperactivity) regulate lung cancer, as well as their synergistic remodeling effects on key TME components (exosomes, tumor-associated macrophage polarization, cancer stem cells, and epigenetic modifications); second, clarifying the molecular regulatory role of lung cancer heterogeneity and identifying mechanism-specificity across different subgroups; third, summarizing potential effective therapeutic agents and subtype-matched clinical strategies based on core molecular targets. This review aims to construct a molecular regulatory network underlying the interaction between OSA and lung cancer, providing solid mechanistic support and practical reference for the precise clinical management of this comorbid condition.

## 2 Molecular mechanisms of OSA-mediated lung cancer

### 2.1 CIH-mediated TME modifications and related signaling pathways

#### 2.1.1 Tumor microenvironment

The TME is a dynamic environment made up of various cellular players, including fibroblasts, immune cells, inflammatory cells, epithelial cells, endothelial cells, mesenchymal cells, adipocytes, and extracellular matrix (ECM) (Ping et al., 2021). The substantial reciprocal communications between altered tumor cells and the dynamic TME establish pathways and signalling networks that drive tumorigenesis and cancer progression (Yuan et al., 2022). Growing evidence has shown that exposure to CIH fosters the development of stable genotypic and phenotypic traits, which can significantly enhance tumor advancement (Verduzco et al., 2015) and modulate the communication between tumor cells and stromal cells via the COX-2/PGE2 signaling pathway (Picado and Roca-Ferrer, 2020). Meanwhile, CIH may affect cytokine levels in the TME, such as tumor necrosis factor-α (TNF-α), interleukin-8 (IL-8), and interleukin-6 (IL-6), through the activation of NF-κB (Li et al., 2011). Ma et al. observed that CIH exposure lowers interleukin-17 (IL-17) level while boosting the levels of interleukin-10 (IL-10) and TNF-α within solid tumors, which facilitates tumor immune evasion within the TME, thus fueling tumor progression and malignancy (Ma et al., 2021). In summary, CIH may enhance solid tumor progression and metastasis by modulating the tumor’s inflammatory microenvironment and immune response.

Research also indicates that obesity is closely associated with multiple types of cancer, particularly lung cancer. The biological mechanisms linking obesity to lung cancer risk are multifaceted, potentially involving complex interactions between adipose tissue, systemic chronic inflammation, and insulin resistance. Firstly, adipose tissue leads to the secretion of pro-inflammatory cytokines and adipokines, such as TNF-α, IL-6, and leptin, thereby creating a TME conducive to cancer progression and metastasis (Liu X. et al., 2023; Lee et al., 2015). Then, excess weight leads to an expansion of adipose tissue, resulting in hypoxia and oxidative stress (Sergeeva et al., 2023). Reactive oxygen species (ROS) brought on by CIH directly damage DNA, inducing gene mutations, and further activate inflammatory pathways, such as the promotion of TNF-α and IL-6 release, forming a synergistic effect with obesity-related chronic inflammation (Ekin et al., 2021; Lamabadusuriya et al., 2025). Finally, Liu J. et al. found that insulin resistance is significantly positively correlated with the risk of lung cancer (Liu et al., 2024). The underlying mechanism may involve abnormal expression of proteins such as the insulin-like growth factor 1 receptor (IGF-1R) and the insulin receptor substrate 2 (IRS2), and the abnormal activation of these molecules may be implicated in the growth regulation of lung cancer cells (Shahid et al., 2024; Piper et al., 2019). Overall, targeting adipocytes, cytokines, and their signaling pathways presents a promising strategy for cancer treatment.

Cancer-associated fibroblasts (CAFs) are among the most important players in the TME and are activated fibroblasts exhibiting remarkable adaptability and heterogeneity in the TME, with diverse functions in tumor initiation, progression, invasion, and metastasis that have been extensively studied (Ping et al., 2021; Biffi and Tuveson, 2021). However, the lack of well-characterised markers and a uniform terminology to describe different phenotypes is a major obstacle to understanding the biological characteristics of CAFs. Recent research has specifically highlighted their role in identifying distinct tumor cell features and microenvironment compartments that displayed remarkable heterogeneity and dynamic changes in response to treatment, by using technological advances, such as single-cell RNA-sequencing (scRNA-seq) (Yan et al., 2025; Cords et al., 2024). Interestingly, recent studies indicate that CIH accelerates the advancement of lung cancer by boosting the mobility of lung cancer cells through upregulating transforming growth factor β (TGF-β) signaling, thereby increasing the activation and proportion of lung CAFs (Cui et al., 2023). Thus, we believe that CAFs would be targeted more precisely following the advances in cRNA-seq and provide therapeutic benefits for OSA-associated lung cancer patients.

It has been reported that hypoxia triggers angiogenesis and metastasis. Hypoxic conditions stabilize hypoxia-inducible factor-1 (HIF-1), thereby promoting angiogenesis and metabolic reorganization (Fu et al., 2021). Furthermore, hypoxia triggers a substantial buildup of lactate, creating an acidic setting. Within the TME, this low pH compromises the effectiveness of cytotoxic CD8^+^ T cells and natural killer (NK) cells, while simultaneously amplifying the immunosuppressive activity of CAFs. Meanwhile, these conditions can also promote cancer cell proliferation (Boedtkjer and Pedersen, 2020). This may be a potential mechanism by which OSA leads to lung cancer progression.

#### 2.1.2 Exosome and its carried miRNA

Exosomes are vesicular mediators equipped with a lipid bilayer structure that carry biomolecules such as signal transduction proteins, metabolites, and microRNAs (miRNAs) to transmit signals between cells. Serving as key regulatory carriers in the progression of OSA-associated lung cancer, they modulate tumor cell proliferation, drug resistance, and immune responses within the TME (Jeppesen et al., 2019). CIH, a core pathophysiological feature of OSA, markedly promotes exosome secretion from tumor cells. These exosomes not only enhance the malignant phenotype of tumor cells and disrupt endothelial barrier integrity but also exert pro-tumor effects that are reversible via CPAP treatment (Almendros et al., 2016; Khalyfa et al., 2018a; Khalyfa et al., 2018b). Additionally, hypoxia-induced exosomes have been shown to drive the progression of lung cancer, as well as breast cancer, prostate cancer, and other malignancies (King et al., 2012; Ramteke et al., 2015; Kucharzewska et al., 2013), by regulating angiogenesis, stemness, and other processes (Ramteke et al., 2015; Kucharzewska et al., 2013; Ren et al., 2024).

miRNAs encapsulated in exosomes are core molecules regulating lung cancer, among which miR-210 and miR-106a-5p have the most well-defined mechanisms. miR-210 is upregulated by hypoxia-inducible factor-1α (HIF-1α) under CIH conditions, and synergistically regulates key processes such as angiogenesis, epithelial-to-mesenchymal transition (EMT), and therapy resistance by targeting downstream molecules including PTPN1 (enhancing immune escape), SDHD (inducing mitochondrial metabolic reprogramming), and E2F3 (promoting lung adenocarcinoma development) (Goyal et al., 2025; Zhang et al., 2024). miR-106a-5p induces M2 polarization of macrophages by inhibiting PTEN or activating the STAT3 signaling pathway, thereby enhancing the proliferation and invasion abilities of non-small cell lung cancer (NSCLC) (Ren et al., 2024).

Other differentially expressed miRNAs mainly function in a synergistic network: 11 differentially expressed miRNAs, such as mmu-miR-671-5p, mmu-miR-609, mmu-miR-5113, and others, identified in exosomes from CIH-exposed mice are primarily involved in post-transcriptional modification, protein synthesis, cell morphology, cellular compromise, and other functions (Almendros et al., 2016). Additionally, hypoxia can also inhibit miR-27a expression via the NRF2 pathway, relieving its inhibition on BUB1 and facilitating lung cancer cell proliferation and EMT (Liu C. et al., 2023). Moreover, exosomes derived from NSCLC patients with OSA can directly promote PD-L1 expression in macrophages, enhancing immunosuppressive effects (Liu Y. et al., 2022).

Given the excellent stability and detectability of exosomal miRNAs in body fluids, these molecules not only provide critical insights into the interaction mechanisms between OSA and lung cancer but also hold promise as non-invasive biomarkers for early disease diagnosis and therapeutic monitoring, offering new directions for clinical precision intervention.

#### 2.1.3 TAMs and macrophage polarity

Tumor-associated macrophages (TAMs) are a kind of innate immune cell in cancers. M1-M2 polarization axis is associated with cancer progression; M1 phenotype macrophages are tumor-resistant and can enhance anti-tumor inflammatory reactions, while the M2 phenotype has tumor-promoting capabilities involving angiogenesis, immunosuppression, and neovascularization, as well as stromal activation and remodeling (Malfitano et al., 2020). Both adipose tissues (AT) around the tumor and bone marrow (BM)-derived progenitor cells represent potential macrophage sources (Wagner et al., 2012). In addition, the interactions between various cells could result in macrophage function changes in the TME (Wang et al., 2022).

In the mouse model of lung cancer, Almendros et al. revealed that CIH could regulate the interaction between tumor cells and AT, promote the transformation into adipose tissue macrophages (ATMs), and increase BM-derived ATMs. Furthermore, the M2/M1 ratio increased in IH mice, showing that the TAMs may experience a polarity shift toward an activated M2 macrophage phenotype. Thus, macrophages may be derived from adipose tissues surrounding the tumor, which undergo polarity shifts from M1 towards M2 phenotype, migrate into the tumor, become a part of the TME, proliferate *in situ*, and, in turn, promote tumor growth, invasiveness, proliferation, and EMT finally (Almendros et al., 2015; Almendros et al., 2014). The aforementioned studies also indicate that obesity-related chronic inflammation and local hypoxia may promote tumor progression via pathways similar to CIH-adipose tissue-TME. Therefore, CIH could change TME and influence OSA-associated lung cancer by macrophage polarity, exosome secretion, and immune escape.

#### 2.1.4 Cancer stem cells (CSCs)

CSCs are a subpopulation of cancer cells with self-renewal capacity, playing critical regulatory roles in tumorigenesis, tumor growth, recurrence, metastasis, EMT, and therapeutic responses (Fu et al., 2017). Cells with CSC potential can be identified via antibody-based sorting, tumor sphere formation, and other techniques (Nguyen et al., 2012), and these cells are capable of metastasizing from the primary site to distant organs to initiate metastatic tumors (Eramo et al., 2008; Dzobo et al., 2020). In lung cancer, CSCs regulate core signaling pathways including Janus kinase (JAK)/signal transducer and activator of transcription (STAT), nuclear factor κB (NF-κB), Notch, PI3K/AKT serine/threonine kinase, SHH, and Wnt/β-catenin, while also participating in TME remodeling and cancer drug resistance mechanisms (Heng et al., 2019).

In addition, studies showed that CSCs have an important role in the condition of CIH (Peng and Liu, 2015). Hao et al. demonstrated that CIH promotes lung cancer stemness by activating mitochondrial ROS (mtROS), a process partially mediated by CNC homolog 1 (Bach1) (Hao et al., 2021). Subsequent validation by the same research team revealed that CIH-induced upregulation of HIF-1α/ATAD2 exerts a significant regulatory effect on lung cancer stemness (Hao et al., 2022). Akbarpour’s team also confirmed that CIH enhances the expression of stemness markers, endowing tumors with stronger metastatic potential (Akbarpour et al., 2017). Additionally, CIH activates EMT-related transcription factors and promotes the dedifferentiation of cancer cells into CSCs by upregulating embryonic stem cell transcription factors such as OCT4, SOX2, and NANOG (Díaz-et al., 2021). Previous studies have also shown that CIH mediates the acquisition of CSC characteristics by regulating the TGF-β signaling pathway via paraspeckle component 1 (PSPC1) (Pengo et al., 2021).

At the immunoregulatory level, CIH-induced CSC-like properties are closely associated with impaired anti-tumor immune function: CIH upregulates the PD-1/PD-L1 pathway to inhibit CD8^+^ T cell cytotoxicity. Although CIH increases the number of CD8^+^ T cells, these immune cells—normally secreting granzyme B and perforin—exhibit impaired function, thereby promoting tumor growth and invasion (Akbarpour et al., 2017; Cubillos-Zapata et al., 2017). However, this mechanism remains controversial: Recoquillon et al. found that OSA did not alter exosomal PD-L1 expression or peripheral lymphocyte activity (Recoquillon et al., 2024), with core differences attributed to variations in PD-L1 expression carriers, immune cell targets, OSA pathophysiological features (CIH alone vs. combined with SF), and age stratification across studies. Future research should unify the detection dimensions of PD-L1 expression and immune cells, and conduct integrated analyses incorporating OSA pathophysiological characteristics and age factors to clarify the specific regulatory mechanism of this association.

Drug resistance is also closely linked to CIH-induced CSC-like properties. Gu et al. constructed a lung cancer stem cell (LCSC) model and a mouse model with tumors *in situ* and found that CIH exposure leads to cisplatin resistance; the tumor spheres formed from NSCLC cell lines were also identified in this condition (Gu et al., 2021). Except for cisplatin resistance, CIH directly induces resistance to doxorubicin in NSCLC (Song et al., 2006). Chemotherapy is a potential method in the therapy of lung cancer, but acquired cisplatin resistance has become a serious and primary problem in this course of treatment. Therefore, CSCs and their related pathway signals may be a new therapeutic target in treating OSA-associated lung cancer and chemoresistant OSA-associated lung cancer.

#### 2.1.5 m^6^A RNA methylation and DNA methylation

N6-methylation (m^6^A) is the most common internal modification found in mRNA within higher eukaryotes (Pan, 2013). The process is carefully managed by methylases (writers), demethylases (erasers), and proteins that recognize methylation (readers). Recent research has indicated that m^6^A modification could influence cell proliferation, metastasis, apoptosis, and cell death in cancers; thus, m^6^A has been proposed as a novel therapeutic and diagnostic target in cancers (Mobet et al., 2022; Khan and Malla, 2021). For example, overexpression of ALKBH5 (human AlkB homolog H5) could decrease the expression of cellular m^6^A level (Zheng et al., 2013), which belongs to the AlkB family of iron (II)/α-ketoglutarate (α-KG)-dependent dioxygenases (Kurowski et al., 2003).

At present, the role and mechanism of m6A modification in the occurrence and development of cancer are one of the hotspots in tumor biology research. m6A modification plays a pivotal role in the occurrence and development of lung cancer. Also, recent studies suggested that methylation could cause hypoxia in OSA. Chao et al. found increased ALKBH5 in lung cells (A549 and HCI-H522) under the condition of intermittent hypoxia (64 cycles of 5 min sustained hypoxia [1% O_2_, 5% CO_2_, and balanced N_2_] and 10 min normoxia) compared with RA, while its inhibition repressed the growth and invasion of lung cancer under CIH; during this process, m6A levels were increased and FOXM1 was decreased, which suggests that ALKBH5 regulates FOXM1 mRNA modification and translation (Chao et al., 2020). Another study found that m6A demethylase ALKBH5 downregulates m6A modification on FOXM1 mRNA and promotes FOXM1 expression in patients with OSA-associated lung cancer, which could be a new sight for the diagnosis and treatment of patients with OSA-associated lung cancer.

DNA methylation is also a type of epigenetic modification that has an important role in respiratory diseases, such as lung cancer and OSA (Al-Yozbaki et al., 2022; Zhang et al., 2019). Cortese et al. found that exposure to CIH in mice enhances the release of circulating DNA into circulation, which carries distinctive epigenetic signatures that may characterize cell populations within the tumor that are more prone to shedding their DNA under CIH conditions (Cortese et al., 2015).

#### 2.1.6 Related signaling pathway

**FIGURE 1:**
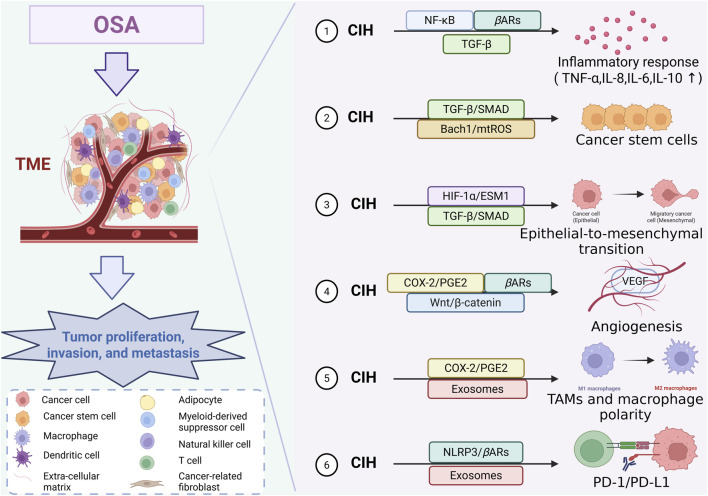
Infographic illustrating how OSA affects the tumor microenvironment (TME) and promotes lung cancer proliferation, invasion, and metastasis. Six key pathways mediated by chronic intermittent hypoxia (CIH) are shown, including inflammation, cancer stemness, EMT, angiogenesis, macrophage polarization, and immune checkpoint regulation. A cell legend labels the main cellular components in the TME. OSA, obstructive sleep apnea; CIH, chronic intermittent hypoxia; TME, tumor microenvironment; TAMs, tumor-associated macrophages (Created with BioRender.com).

Some related factors, such as endothelial cell-specific molecule-1 (ESM1), HIF‐1α, vascular endothelial growth factor (VEGF), Bach1, COX-2, PGE2, and other factors, participated in this process. Several studies have explored the following pathways concerning the relationship between OSA and lung cancer, revealing the importance of CIH in the OSA state, affecting the development of lung cancer (Figure 1).

##### 2.1.6.1 HIF‐1α and VEGF

Most experiments suggested that HIF‐1α is overexpressed in OSA-associated lung cancer (Cubillos-Zapata et al., 2019a). In a case series, the authors calculated several cases over 5 years and showed that severe OSA heightened cancer mortality risk in stage III-IV lung cancer patients, correlating strongly with HIF-1α overexpression (Huang et al., 2020). HIF serves as a key regulator of VEGF during hypoxia in lung cancer cell lines, where elevated HIF-1 expression promotes angiogenesis in response to hypoxia (Abolfathi et al., 2021). Actually, not all experiments demonstrated that HIF‐1α could be upregulated in the condition of CIH in a lung cancer mouse model. Kang et al. evaluated whether OSA-related chronic CIH affects lung cancer progression and elucidated the underlying mechanisms in a murine lung cancer model. They found that CIH enhances proliferative and migratory properties of tumors and that β-catenin and Nrf2 appeared to be crucial mediators of tumor growth (Kang et al., 2020). Wnt/β-catenin signaling could promote carcinogenesis (Polakis, 2012) in lung cancer, hepatocellular carcinoma, and other various types of cancers (Huang F. et al., 2019; Hong et al., 2017; Luo et al., 2019; Choi et al., 2010). Interestingly, Nrf2 can either inhibit or promote cancer progression depending on the cell type; it is regarded as a tumor suppressor due to its cytoprotective functions. Yet, it could also be a helper in creating a favorable intracellular environment for tumor cells to thrive, thereby potentially fueling the growth of tumors (DeNicola et al., 2011; Menegon et al., 2016).

VEGF is overexpressed in lung cancer. Studies have shown that VEGF expression is positively correlated with a higher stage of lung cancer progression (Tian et al., 2020; Li et al., 2020). Some studies found higher expression of VEGF in the plasma of patients with OSA + lung cancer group compared to those with OSA or lung cancer alone (Liu et al., 2020). The results were consistent with Zhang et al. (2019) and Kang et al. (2020). Yet, the detailed mechanism of VEGF function in OSA-associated lung cancer is still not fully understood.

##### 2.1.6.2 COX-2/PGE2

Cyclooxygenase-2 (COX-2) catalyzes the rate-limiting step in the production of eicosanoids. COX-2 could be rapidly and highly inducible at the site of inflammation (Crusz and Balkwill, 2015). Precursor molecule prostaglandin H_2_ (PGH_2_) is a member of numerous metabolites formed from arachidonic acid. PGH_2_ is also a major player in regulating inflammatory responses by regulating numerous signaling pathways (Wang and Dubois, 2010). COX-2 is overexpressed in lung cancer and associated with cancer proliferation, metastasis, and other biological behaviors (Picado and Roca-Ferrer, 2020; Roca-Ferrer et al., 2011). Studies found that COX-2 could induce the overexpression of VEGF (Xue and Shah, 2013), and the COX-2/PGE_2_ pathway has a significant role in the angiogenesis of cancers, as well as in the condition of CIH (Xue and Shah, 2013; Lee et al., 2010). Campillo et al. found that CIH could mediate the malignancy of lung cancer through the COX-2/PGE_2_ signaling pathway; they established lung carcinoma tumor mouse models with or without the condition of CIH and showed that CIH may enhance tumor progression both directly and via host immune alterations. CIH could also activate the COX-2 pathway and PGE_2_, and accelerate tumor progression with TAMs shift from M1 towards M2 phenotype. The inhibitor of COX-2 could suppress PGE_2_ production *in vitro* (Campillo et al., 2017). In conclusion, under the condition of CIH, the COX-2/PGE2 signaling pathway plays a critical role in both tumor cells and macrophages. While preclinical studies in murine models have provided initial evidence of its pro-tumorigenic effects, these findings still require validation in large-scale clinical cohorts to clarify the pathway’s actual relevance and translational potential in humans.

##### 2.1.6.3 βARs

The β-adrenergic receptors (βARs) constitute a subfamily of G protein-coupled receptors (GPCRs) that are categorized into three distinct subtypes: β_1_-, β_2_-, and β_3_ (Velmurugan et al., 2019). As critical effectors of the sympathetic nervous system, βARs bind to catecholamines—core neurotransmitters released upon sympathetic activation—to regulate not only basic physiological processes such as cardiac performance, airway responsiveness, and metabolic balance (Insel, 1996) but also exert a pivotal regulatory role in tumorigenesis and progression. Cumulative evidence confirms that βARs are weakly expressed in normal tissues yet abnormally overexpressed in malignant tumors, including lung, colorectal, and breast cancer (Rains et al., 2017). Notably, both cancer cells and immune cells in the TME often co-express βARs, laying a molecular foundation for their regulatory role in tumor biology (Eng et al., 2014).

CIH, a core pathophysiological feature of OSA, can continuously activate the sympathetic nervous system to release catecholamines, thereby triggering the βARs signaling pathway and promoting lung cancer progression (Powell et al., 2013; Cui et al., 2021). Three key molecular mechanisms drive this effect: first, catecholamine binding to βARs upregulates vascular endothelial growth factor receptor 2 (VEGFR2), whose subsequent interaction with VEGF initiates downstream PI3K phosphorylation cascades, fueling tumor angiogenesis to support cancer cell proliferation and invasion; second, it establishes an immunosuppressive microenvironment by upregulating the PD-1/PD-L1 pathway and triggering inflammatory response networks, facilitating tumor immune escape; third, it directly accelerates lung cancer cell proliferation and enhances their malignant phenotype (Sun et al., 2024).

Given the critical role of βARs signaling in CIH-induced lung cancer, β-blockers hold promise as potential therapeutics for OSA-associated lung cancer (Mas and sagué, 2012). However, most supporting evidence stems from preclinical studies, and their clinical safety, efficacy, and applicable populations in humans remain unclear. Future research demands multi-center, large-scale clinical trials that integrate OSA severity (e.g., CIH frequency, hypoxia duration) and lung cancer subtype characteristics to systematically validate the clinical value of β-blockers, thereby providing a novel strategy for the precise treatment of OSA-associated lung cancer.

##### 2.1.6.4 TGF-β

TGF-β, a multifunctional pro-inflammatory cytokine, regulates various physiological and pathological processes during normal development and tumorigenesis, and displays a distinct “dual role”: it acts as a tumor suppressor in early-stage tumors but shifts to a pro-tumorigenic role in advanced disease (Mas and Sagué, 2012). The key mechanism driving this functional shift is tied to the hypoxic TME: hypoxia in advanced solid tumors induces upregulation of HIF-1α, which binds Smad3 to form a transcriptional complex. This Smad-HIF-1α complex alters Smad3’s binding partners, thus abrogating TGF-β-driven suppression of c-Myc and ultimately promoting tumor progression (Huang et al., 2021). Additionally, TGF-β accelerates tumor progression by suppressing immune surveillance, promoting angiogenesis, and inducing EMT, with pathway activation consistently linked to poor prognosis in lung cancer patients (Miyazono et al., 2012; Kim et al., 2020; Kong et al., 1999).

CIH, a core pathophysiological feature of OSA, acts as a key trigger for TGF-β signaling and lung cancer progression, via two core regulatory mechanisms: first, CIH directly activates HIF-1α, subsequently upregulating TGF-β expression and driving Smad phosphorylation to induce myofibroblast differentiation and extracellular matrix (ECM) production (Liang et al., 2024); second, CIH upregulates PSPC1, further activating the TGF-β-Smad pathway and driving the acquisition of EMT and cancer stem cell-like characteristics in lung cancer cells (Kim et al., 2020; Cubillos-Zapata et al., 2023; Hernández-Jiménez et al., 2017).

CIH-activated TGF-β signaling accelerates lung cancer development through multi-faceted regulation of the TME and tumor cell phenotype: ① it boosts the migratory capacity of lung cancer cells, activates CAFs, and increases their numbers—CAF-ECM crosstalk is pivotal for tumor invasion (Cui et al., 2023); ② it centrally governs ECM remodeling by stimulating the production of hyaluronan synthases (HAS1-3) and hyaluronidases, simultaneously promoting ECM stiffening and degradation to increase tumor rigidity and facilitate metastasis (Sapudom et al., 2020); ③ it disrupts cytokine network homeostasis by upregulating TNF-α and IL-10 while downregulating IL-17, impairing anti-tumor immunity and creating an immunosuppressive environment for tumor growth (Ma et al., 2021).

Given the critical mediating role of TGF-β in CIH-induced lung cancer, inhibiting TGF-β signaling stands as a highly promising therapeutic approach—studies confirm that targeting TGF-β reduces collagen levels in the lung tumor ECM and disrupts pro-tumorigenic CAF-ECM interactions (Horie et al., 2014). While the exact molecular mechanisms through which TGF-β drives lung cancer progression remain incompletely defined, this pathway provides a key target for novel targeted therapies against OSA-associated lung cancer, laying the groundwork for clinical translational research.

##### 2.1.6.5 Wnt/β-catenin

The Wnt/β-catenin signaling pathway is an evolutionarily highly conserved core pathway, activated when Wnt proteins bind to cell surface receptors via autocrine or paracrine signaling. This activation initiates a cascade reaction that maintains β-catenin stability and promotes its nuclear translocation, thereby regulating the expression of genes involved in cell development, multiplication, specialisation, and programmed cell death (Zhao et al., 2024; Ma et al., 2023; Cruciat and Niehrs, 2013). Notably, dysregulation of this pathway is linked to a range of diseases, most notably malignant tumors such as lung cancer (Teng et al., 2010; Malyla et al., 2023), breast cancer (Wellenstein et al., 2019), and colorectal cancer (Morin et al., 1997), while also contributing to the pathogenesis of non-cancer diseases, including chronic obstructive pulmonary disease (Skronska-Wasek et al., 2017) and atherosclerosis (Döring et al., 2017).

In lung cancer, β-catenin is a key molecule governing tumor growth and EMT. Studies have confirmed that inhibiting the Wnt/β-catenin pathway effectively blocks lung cancer pathogenesis and slows disease progression (V et al., 2012). Hypoxia, a core feature of the TME, engages in complex crosstalk with the Wnt/β-catenin pathway, significantly impacting cancer malignancy and metastatic potential. Central to this interaction are HIF family members, which serve as key mediators: HIF-1α amplifies Wnt/β-catenin signaling in hypoxic environments by enhancing β-catenin activity and upregulating downstream targets such as LEF-1 and TCF-1 (Mazumdar et al., 2010); meanwhile, HIF-2α overexpressed in lung cancer cells directly boosts β-catenin expression, promoting tumor cell migration and invasion (Hong et al., 2017). CIH, a core pathophysiological feature of OSA, reinforces this regulatory network. Kang et al. validated in a lung cancer mouse model that CIH directly triggers the activation of Wnt/β-catenin pathway-related genes and promotes β-catenin nuclear translocation (Kang et al., 2020), ultimately driving lung cancer progression. Given the critical driving role of the Wnt/β-catenin pathway in CIH-induced lung cancer, blocking this pathway holds promise as a potential therapeutic strategy to slow the progression of OSA-associated lung cancer.

##### 2.1.6.6 NF-κB

Nuclear factor-κB (NF-κB) is a key family of transcription factors, named for binding to the regulatory region of the immunoglobulin κ light chain-encoding gene in B cells (Sen and Baltimore, 1986). Its canonical pathway is a core mechanism mediating inflammatory responses and is closely associated with tumorigenesis and disease progression (Prasad et al., 2010). CIH—a core pathophysiological feature of OSA—induces hypoxia-reoxygenation cycles that trigger oxidative stress and systemic inflammation, leading to excessive production of ROS (Jelska et al., 2024). As a key ROS-inducible transcription factor, NF-κB acts as the central link between oxidative stress and inflammatory responses (Scholz and Taylor, 2013).

CIH activates the NF-κB pathway through multiple mechanisms, and its core regulatory network centers on crosstalk between HIF-1α and ROS: on one hand, CIH upregulates HIF-1α levels in a ROS-dependent manner, while HIF-1α is also critical for CIH-induced ROS generation, forming a positive feedforward loop (Prabhakar and Semenza, 2012); on the other hand, under hypoxic conditions, HIF-α subunits interact with prolyl hydroxylases (PHDs), alleviating PHD-driven inhibition of IKKβ and partially activating the canonical NF-κB pathway (Cummins et al., 2006). Additionally, HIF-1α overexpression can directly stimulate NF-κB activity to potentiate the inflammatory cascade (Scortegagna et al., 2008), and the abnormal upregulation of inflammatory molecules and cytokines further promotes lung cancer cell proliferation, invasion, and metastasis (Tang et al., 2025; Germano et al., 2008).

Sustained activation of the NF-κB pathway fuels lung cancer progression through multiple avenues: it remains constitutively active in lung cancer stem cells, acting as a key regulator of their proliferation, survival, and stemness maintenance (Rinkenbaugh et al., 2016; Zakaria et al., 2018); proteomic studies confirm its involvement in core biological processes of lung cancer, including inflammatory signaling, metabolism of oxidative stress intermediates, and gluconeogenesis/glycolysis (Pastor et al., 2013). Furthermore, a study demonstrated that CIH can induce the upregulation of multiple pro-metastatic genes by activating the NF-κB pathway in inflammatory breast cancer cells (Gutsche et al., 2016), highlighting the universal relevance of this mechanism across CIH-associated tumors. Given NF-κB’s critical mediating role in CIH-induced lung cancer, it holds promise as a potential molecular therapeutic target for OSA-associated lung cancer.

##### 2.1.6.7 PD-1/PD-L1 axis

Programmed cell death 1 (PD-1) and its ligand (PD-L1) are key molecules of the immune system’s core inhibitory pathway, with their mediated immune escape being a key driver of tumor progression: malignant tumor cells highly express PD-L1 on their membranes, binding to T cell surface PD-1 to form a complex that directly inhibits cytotoxic T lymphocyte proliferation and cytotoxicity, enabling tumor immune evasion (Cascone et al., 2022). In lung cancer, high PD-L1 expression is closely associated with poorer progression-free survival (PFS), overall survival (OS), and lower objective response rate (ORR) (Gandhi et al., 2018; Socinski et al., 2018), highlighting the pathway’s critical role.

CIH—a core pathophysiological feature of OSA—regulates the PD-1/PD-L1 pathway through multiple dimensions to enhance lung cancer immune escape: ① Direct regulation of cell surface molecules: Cubillos-Zapata et al. confirmed PD-L1 overexpression in peripheral blood monocytes of OSA patients; CIH induces PD-L1 upregulation in monocytes from healthy volunteers, while enhancing PD-1 expression on CD8^+^ T cells and recruiting myeloid-derived suppressor cells (MDSCs) to establish an immunosuppressive network (Cubillos-Zapata et al., 2017). Animal experiments show CIH promotes tumor growth (increased volume and weight) in lung cancer mice, which correlates positively with PD-L1 expression (Huang M. H. et al., 2019). ② Exosome-mediated indirect regulation: Liu Y. et al. found exosomes secreted by lung cancer cells under CIH conditions enhance macrophage PD-L1 expression via the HIF-1α pathway, amplifying immunosuppression (Liu Y. et al., 2022). ③ Regulation of soluble PD-L1: OSA-related hypoxemia increases PD-1/PD-L1 levels in lung cancer patients and high-risk populations, while upregulating soluble PD-L1 to further impair anti-tumor immunity (Cubillos-Zapata et al., 2024). Additionally, a multicenter study of 360 patients showed elevated PD-L1 expression in cutaneous melanoma of severe OSA patients (Cubillos-Zapata et al., 2019b), indirectly confirming the cross-tumor universality of CIH’s regulation on this pathway.

Based on the PD-1/PD-L1 pathway’s core role, PD-1/PD-L1 inhibitors are established as a key therapeutic strategy for lung cancer—they block PD-1/PD-L1 binding, restore T cell recognition and tumor cell clearance, delay tumor growth, and prolong survival. Recent studies further demonstrate that Sema4A expressed by tumor cells in the TME activates tumor-infiltrating CD8^+^ T cells, significantly enhancing the sensitivity of mouse models and NSCLC patients to PD-1 blockade (Naito et al., 2023), offering a novel avenue to optimize therapeutic regimens. Given CIH’s regulatory role, these inhibitors are expected to improve lung cancer treatment outcomes under hypoxic microenvironments, serving as a potential effective strategy for OSA-associated lung cancer. While existing evidence confirms the association between CIH and the PD-1/PD-L1 pathway, critical gaps remain: the specific molecular mechanisms of PD-1/PD-L1 expression mediated by different cell types and the dose-effect relationship between CIH severity and PD-L1 expression. Future targeted studies are needed to dissect core regulatory nodes, providing a solid theoretical basis for optimizing immunotherapeutic regimens for OSA-associated lung cancer.

##### 2.1.6.8 Other factors

In addition to the well-known pathways mentioned above, several novel targets have been identified by researchers in recent years. The following therapeutic targets hold promise as potential treatment options for OSA-associated lung cancer (Table1).

**TABLE 1:** Other therapeutic targets/pathways for OSA-associated lung cancer.

Therapeutic target	Experimental subject	Promote/inhibit tumor growth	Mechanism	References
Follistatin-like 1 (Fstl1)	C57BL/6 mice	Inhibit tumor growth	Decrease the expression of HIF-1α, VEGF, and EMT	Qi C. et al. (2023)
Endothelial cell-specific molecule-1 (ESM1)	Human lung cancer cells (PC-9 and A549 cells)	Promote tumor growth	Induced by HIF-1α to increase the expression of EMT	Gu et al. (2021)
BTB and CNC homology 1 (Bach1)	C57BL/6 male mice	Promote tumor growth	Increase the expression of mtROS and increasee CSC-related translators and markers	Hao et al. (2021)
Nucleotide-binding oligomerization domain-like receptor protein 3(NLRP3)	C57BL/6 N male mice	Promote tumor growth	Upregulation of PD-1 and PD-L1	Sun et al. (2024)
Paraspeckle component 1 (PSPC1)	Patients	Promote tumor growth	The activation of the TGFβ/SMAD pathway, promoting EMT and CSC	Díaz- et al. (2021)
ATPase family AAA domain-containing 2 (ATAD2)	Lung adenocarcinoma tissues and matched adjacent tissues	Promote tumor growth	Increase mtROS levels	Hao et al. (2022)

EMT, epithelial-to-mesenchymal transition; mtROS, mitochondrial ROS; CSC, cancer stem cell.

### 2.2 SF-mediated lung cancer progression: core mechanisms and synergistic amplification with CIH

SF, a hallmark pathophysiological feature of OSA, is characterized by recurrent arousals causing sleep discontinuity (Zhang et al., 2014; Hakim et al., 2014). It directly drives lung cancer progression through multi-dimensional independent mechanisms and acts as a key synergistic factor to amplify the pro-carcinogenic effects of CIH, with core regulation focusing on immune microenvironment remodeling, oxidative stress, intercellular communication, and neuro-inflammatory crosstalk (Gozal et al., 2015). Although direct human epidemiological evidence is currently lacking, numerous animal and cellular experiments have confirmed its important role in OSA-associated lung cancer.

#### 2.2.1 Immune microenvironment remodeling: TAMs polarization and TLR4 signaling activation

SF constructs an immunosuppressive microenvironment to directly promote lung cancer progression by regulating the function of TAMs and downstream signaling pathways. Studies have shown that SF can recruit TAMs and induce their polarization toward the pro-tumor M2 phenotype, while upregulating TLR4 expression on TAMs and activating the TLR4 signaling pathway (partially involving MYD88 and TRIF pathways) (Hakim et al., 2014). This process enhances matrix metalloproteinase (MMP) activity, accelerates lung cancer cell proliferation and invasion, and increases serum TNF-α levels to further strengthen immunosuppression (Xian et al., 2021). Additionally, SF downregulates the expression and activity of gp91phox in TAMs, reducing NADPH oxidase 2 (Nox2) activity and ROS production, impairing the anti-tumor function of TAMs and indirectly promoting tumor progression (Zheng et al., 2015).

#### 2.2.2 Oxidative stress and DNA damage: directly driving lung cancer initiation

SF induces oxidative stress by increasing tissue ROS levels, leading to DNA damage, and this effect is independent of enhanced inflammation. Research in colorectal cancer models has confirmed that SF can increase ROS content in colon tissue, resulting in 8-OHdG-related DNA damage, thereby increasing tumor number and enlarging tumor volume (Lee D. B. et al., 2023). This mechanism is also potentially applicable in lung cancer, suggesting that SF may provide an initial driving signal for lung cancer initiation by directly disrupting genomic stability.

#### 2.2.3 Exosome-mediated intercellular communication: transmitting pro-tumor signals

SF mediates the transmission of pro-tumor signals between cells by altering the molecular cargo of circulating exosomes. Khalyfa et al. found that circulating plasma exosomes obtained from SF mice or OSA patients can enhance the proliferation and migration abilities of tumor cell lines (Khalyfa et al., 2016). Further studies have shown that SF exposure can induce mice to produce exosomes containing unique miRNAs (miR-5128, miR-5112, miR-6366), which can act as molecular carriers to regulate the cell cycle and immune escape of lung cancer cells. In addition, SF can promote the overexpression of lung cancer stem cell markers (CD133, CD44) through exosomes, maintaining tumor stemness and enhancing its malignant potential (Akbarpour et al., 2017).

#### 2.2.4 Multi-system synergistic regulation: gut microbiota and proteome remodeling

SF synergistically promotes the formation of a lung cancer metastatic microenvironment by disrupting multi-system homeostasis. In a murine melanoma model, SF can alter lung tissue proteome expression, involving 43 key regulatory genes such as Lama2, Ptk2, and Grb2, focusing on core pathways including focal adhesion and hormone response. Meanwhile, SF disrupts gut microbiota composition, increases the Firmicutes/Bacteroidetes ratio, and abnormally regulates taxa such as *Bacteroides* and Desulfovibrio, indirectly promoting tumor metastasis (Xian et al., 2021). Additionally, sympathetic hyperactivity induced by SF can regulate inflammatory responses through β-adrenergic receptors, further amplifying pro-carcinogenic effects (Wheeler et al., 2021).

#### 2.2.5 Synergistic amplification effect of SF and CIH

SF and CIH synergistically promote lung cancer progression through complementary mechanisms: CIH centers on hypoxia-related signaling pathways (e.g., HIF pathway), while SF focuses on immune microenvironment remodeling and neuro-inflammatory regulation. Notably, SF does not induce significant upregulation of the PD-1/PD-L1 axis in immune cells, nor does it produce a synergistic effect with CIH on this pathway (Cubillos-Zapata et al., 2020). However, SF can significantly amplify CIH-mediated lung cancer initiation and progression by strengthening immunosuppression, accelerating DNA damage, and promoting intercellular pro-tumor signal transmission. Together, they constitute the dual pathological drivers of OSA-promoted lung cancer.

## 3 Reverse regulatory mechanisms of lung cancer on OSA

### 3.1 Tumor size and location

Multiple studies have demonstrated a significantly higher prevalence of OSA in lung cancer patients compared with the general population (Bhaisare et al., 2022; Dreher et al., 2018). Although relevant mechanistic research remains limited, tumor size and anatomical location are considered key contributing factors. Central-type lung cancer, whose primary lesions are adjacent to critical upper airway regions such as the main bronchi and hilum, causes progressive airway lumen stenosis and increased airflow resistance due to direct compression as the tumor grows or mediastinal lymph node metastasis occurs (Mahmood et al., 2025). Superior vena cava syndrome, a common complication of lung cancer, further exacerbates upper airway stenosis (Zidan et al., 2024; Ito et al., 2000). Such structural stenosis is more likely to induce airway collapse during nighttime sleep, when physiological reductions in airway smooth muscle tone and relaxation of supporting structures collectively trigger or worsen obstructive respiratory events. In addition, pleural effusion resulting from lung cancer progression impairs diaphragmatic function, while tumor invasion of the chest wall or advanced cachexia weakens respiratory muscle strength (Cortiula et al., 2022), leading to decreased airway dilation capacity. During nighttime, respiratory muscles cannot effectively counteract airway collapse, prolonging hypoxia and triggering compensatory arousals that ultimately induce SF.

### 3.2 Systemic inflammation and oxidative stress

Lung cancer and its tumor microenvironment can induce or exacerbate OSA through multiple pathways, with chronic inflammation acting as a pivotal mediator. Lung cancer cells and TAMs secrete substantial amounts of pro-inflammatory cytokines, including IL-1β, IL-6, and TNF-α, which promote OSA onset and progression via dual central and peripheral pathways. At the central level, these cytokines access the central nervous system through the blood-brain barrier or vagus nerve pathway, activating microglia and reactive astrocytes to amplify central inflammatory responses. This disruption of core sleep-wake regulatory circuits—such as the hypothalamic-preoptic area and locus coeruleus—ultimately leads to SF (Walker and Borniger, 2019; Mogavero et al., 2021; Ai et al., 2021); At the peripheral level, pro-inflammatory cytokines exacerbate airway mucosal inflammation and neurofunctional dysfunction (Lin et al., 2017), reduce airway smooth muscle tone (Lam et al., 2019), and combined with lung cancer-related airway structural abnormalities, significantly increase the risk of nighttime airway collapse, inducing or worsening OSA.

### 3.3 Treatment-related side effects

Core treatments for lung cancer encompass surgery, chemotherapy, radiotherapy, immunotherapy, and targeted therapy. These interventions collectively drive the onset and progression of OSA by directly damaging airway structures, disrupting respiratory function, or indirectly triggering inflammation and sleep disturbances. Among surgical approaches, lobectomy/pneumonectomy impairs thoracic integrity or respiratory muscle function, reducing respiratory efficiency and thereby increasing OSA risk—fortunately, postoperative respiratory muscle rehabilitation has gained widespread attention and emphasis in clinical practice (Liu et al., 2021; Abidi et al., 2023). Thoracic radiotherapy induces airway mucosal damage and fibrosis, leading to airway lumen stenosis. When combined with the physiological reduction in airway smooth muscle tone at night, this significantly elevates the risk of airway collapse (Xuan et al., 2024). Chemotherapy not only directly impairs sleep parameters (e.g., decreased sleep efficiency, shortened total sleep time) but also indirectly disrupts sleep structure by inducing symptoms such as fatigue and pain, resulting in compromised sleep quality (Dean et al., 2013). A mouse study further confirmed that chemotherapeutic agents (e.g., methotrexate) can induce SF independently of cancer itself, providing direct evidence for chemotherapy-mediated OSA-related pathological processes (Boyd et al., 2025). Regarding immunotherapy, while current research has not identified a direct statistical association between immune checkpoint inhibitor (ICI) treatment and OSA development, ICI-related immune adverse events (e.g., hypothyroidism, airway inflammation) can indirectly affect airway function or sleep structure, thereby exacerbating OSA (Sillah et al., 2022). Additionally, cancer pain is highly prevalent in patients with advanced lung cancer, causing frequent nocturnal arousals and directly inducing SF (Abernethy, 2008; Abebe et al., 2023). However, clinically used sedative-analgesic drugs (e.g., opioids) inhibit the respiratory center and reduce airway reflex sensitivity, further aggravating sleep-related hypoxia and airway obstruction (Webster and Karan, 2020), ultimately forming a vicious cycle that promotes OSA progression.

## 4 Associations between OSA and different lung cancer types

### 4.1 Histological subtype-specific associations: potential differences between NSCLC and SCLC

Lung cancer is histologically classified into non-small cell lung cancer (NSCLC) and small cell lung cancer (SCLC), with NSCLC accounting for 80%–85% of all cases, mainly including adenocarcinoma, squamous cell carcinoma, and large cell carcinoma (Oser et al., 2015). Potential heterogeneity exists in the associations between different subtypes and OSA, which may be attributed to differences in tumor anatomical location, biological characteristics, and responses to hypoxic microenvironment. Notably, most existing studies have focused on NSCLC, while evidence regarding SCLC remains limited.

#### 4.1.1 Lung adenocarcinoma

As the most common subtype of NSCLC, lung adenocarcinoma mainly grows peripherally. Although it rarely directly compresses the central airways, it is prone to complications such as pleural metastasis and pleural effusion in advanced stages. These complications can indirectly interfere with the stability of respiratory rhythm by reducing thoracic compliance and impairing respiratory muscle contraction efficiency, providing a pathological basis for the occurrence of OSA. A cell experiment has shown that different lung adenocarcinoma cell lines [e.g., H522, H1437 (p53 mutant, EGFR wild-type), H1975 (p53 mutant, EGFR mutant)] exhibit certain differences in proliferation rates when exposed to CIH, suggesting that the hypoxic sensitivity of lung adenocarcinoma may be regulated by driver gene mutation status (Marhuenda et al., 2019). Animal experiments further confirmed that CIH can accelerate lung adenocarcinoma progression by altering components of the TME (Qi P. et al., 2023). However, clinical evidence for the association between the above complications and OSA is still scarce, and more clinical data are needed to support the mechanistic hypothesis.

#### 4.1.2 Lung squamous cell carcinoma

The association between lung squamous cell carcinoma and OSA is driven by dual mechanisms, involving anatomical synergy and biological response differences. Anatomically, as a predominantly centrally located tumor, lung squamous cell carcinoma tends to invade or compress the main airways and mediastinum, causing airway stenosis and airflow limitation. This structural airway damage overlaps with the inherent airway collapse mechanism of OSA, which may not only increase the risk of OSA but also exacerbate its severity. Biologically, the association relies on hypoxia-mediated inflammatory infiltration and angiogenesis. Marhuenda et al. confirmed that the lung squamous cell carcinoma cell line H520 (p53 mutant, EGFR wild-type) exhibits a significantly faster proliferation rate than lung adenocarcinoma cell lines under CIH (Marhuenda et al., 2019). The core mechanism is related to the high dependence of lung squamous cell carcinoma on chemokine signaling pathways—CIH upregulates chemokine expression, enhancing the local invasive capacity of lung squamous cell carcinoma. Currently, there are relatively few specialized studies on this association, and the specific association strength and clinical significance between the two await verification by large-sample clinical cohorts.

#### 4.1.3 SCLC

As a highly malignant neuroendocrine tumor, SCLC is characterized by rapid proliferation and early extensive metastasis, with limited dedicated research on its association with OSA. This subtype often causes local invasive symptoms such as superior vena cava syndrome and recurrent laryngeal nerve palsy, directly leading to mechanical airway stenosis or abnormal respiratory neural regulation; the neuroendocrine factors it secretes may also interfere with the rhythm regulation of the respiratory center, theoretically having a pathological basis for inducing or exacerbating OSA. However, due to the poor prognosis and rapid progression of SCLC, clinical studies mostly focus on tumor treatment and survival, with extremely limited dedicated exploration of its association with OSA, and no direct evidence clarifying the correlation and association strength. In addition, a small-sample study involving 69 lung cancer patients showed that SCLC patients had higher apnea hypopnea index, oxygen desaturation index, and time with oxygen saturation <90% compared with adenocarcinoma and squamous cell carcinoma patients, with the proportion of moderate-to-severe OSA reaching 28%. However, statistical significance was not achieved due to limited sample size (only 8 SCLC patients) (Lee H. et al., 2023); clinical prognostic data also indicated that lung cancer subtype is an independent prognostic factor for OSA-associated lung cancer patients (HR = 1.043, 95% CI: 1.002–2.431, P = 0.038), with SCLC patients having significantly shorter overall survival than NSCLC patients, indirectly suggesting a potential specific association between the two (Liu W. et al., 2022).

### 4.2 Genotype-specific susceptibility: potential regulatory roles of EGFR, KRAS, and ALK mutations

The mutation status of molecular driver genes in lung cancer endows different genotypes with specific responses to OSA-related pathological microenvironments by regulating tumor hypoxia response, signal pathway crosstalk, and inflammatory microenvironment. Meanwhile, adverse reactions of targeted therapy may indirectly affect OSA.

#### 4.2.1 EGFR

EGFR mutation is a key regulatory factor for the sensitivity of lung cancer to OSA-related hypoxia. *In vitro* experiments have confirmed that this genotype of lung adenocarcinoma has no obvious response to OSA-related hypoxia (Marhuenda et al., 2019), possibly due to EGFR mutation impairing HIF-1α-mediated signal transduction. The EGFR signaling pathway can regulate the TGF-β/EMT pathway (Moustakas and Heldin, 2016; Cheng et al., 2022), which may synergize with the pro-invasive effect of OSA-induced TGF-β upregulation, but requires verification by functional experiments. In addition, patients often have high release of inflammatory factors such as IL-6 and TNF-α, which may indirectly increase OSA risk, and adverse reactions related to EGFR inhibitors may disrupt sleep. However, there is no specialized data supporting their direct association or the impact of CIH. OSA has a more significant impact on the malignant progression of EGFR wild-type NSCLC, with the core mechanism being OSA-induced upregulation of the co-expressed gene PDGFB (Wang et al., 2021). High PDGFB expression is closely associated with lymph node metastasis in patients with this genotype (Donnem et al., 2010; Don et al., 2011), suggesting that OSA may exacerbate the malignant phenotype by activating PDGFB-mediated angiogenesis and metastasis pathways.

#### 4.2.2 KRAS

CIH may exacerbate abnormal MAP2K3 gene expression (abnormal methylation of this gene is more common in this genotype (Park et al., 2008)), promoting tumor proliferation, invasion, and drug resistance by regulating pathways such as MAPK, with the mechanism to be verified. Meanwhile, this genotype of lung cancer is characterized by high invasiveness and a strong inflammatory phenotype, with elevated levels of pro-inflammatory factors in the tumor microenvironment, which may disrupt airway stability and increase OSA risk. However, the correlation between the two lacks specialized analysis, and the association strength and mechanism need further verification.

#### 4.2.3 ALK

Accounting for 4%–6% of lung adenocarcinoma (Schneider et al., 2023), the unique stromal reaction and inflammatory characteristics of tumors may indirectly affect airway function, and side effects related to ALK inhibitors may interfere with sleep quality. Currently, there are no clinical or basic studies exploring its association with OSA, which is a complete data gap. Targeted explorations are urgently needed to clarify whether there is a specific association between the two.

### 4.3 Research status and prospects

At present, research on the association between OSA and lung cancer with different genotypes is still limited. Existing evidence is mostly based on *in vitro* cell experiments or bioinformatics analysis, lacking verification by large-sample clinical cohorts and *in vivo* functional experiments. In the future, targeted studies are needed. On the one hand, large-scale clinical studies should be carried out to clarify the association strength and prognostic impact of OSA with lung cancer harboring different driver gene mutations. On the other hand, genotype-specific cell models and animal experiments should be used to analyze the molecular mechanisms underlying heterogeneity, providing a theoretical basis for precise intervention in OSA-complicated lung cancer patients.

## 5 Potential therapeutic drugs targeting OSA-lung cancer mechanisms

### 5.1 Anti-inflammatory agents

Chronic inflammation is a core common pathophysiological mechanism of OSA and lung cancer, providing a crucial theoretical basis for the adjuvant use of anti-inflammatory drugs in OSA-associated lung cancer to slow disease progression. Non-steroidal anti-inflammatory drugs (NSAIDs), the most commonly used clinical anti-inflammatory agents, exert their core effect by inhibiting the COX pathway, reducing levels of inflammatory mediators such as PGE_2_ and thromboxane A_2_ (TXA_2_). This further regulates vascular tone and the inflammatory microenvironment, indirectly interfering with OSA-related multisystem damage (Beaudin et al., 2014).

The COX pathway plays a key role in the association between OSA and lung cancer: TXA_2_ derived from COX-1 is significantly elevated in OSA patients, and non-selective COX inhibitors can reduce the risk of cardiovascular complications by inhibiting COX-1 (Beaudin et al., 2014); selective COX-2 inhibitors can decrease TNF-α levels after CIH (P = 0.009) (Beaudin et al., 2015) but may lead to reduced cerebral blood flow (CBF) (P = 0.01) (Beaudin et al., 2014), indicating dual roles of COX-2 in CIH-related inflammation inhibition and vascular homeostasis regulation. The above studies have confirmed that the COX-2/PGE_2_ pathway is a key molecular pathway mediating OSA-associated lung cancer progression, and COX-2-specific inhibitors can effectively block this process. Among them, celecoxib (a 1,5-diarylpyrazole compound), the first COX-2-specific inhibitor used to inhibit tumor apoptosis, proliferation, angiogenesis, and metastasis (Masferrer et al., 2000; Wei et al., 2004), has been shown to suppress CIH-induced malignant transformation of tumors and reverse the abnormal regulation of host immune responses by CIH in mice with OSA-associated lung cancer (Campillo et al., 2017). Its mechanism may involve regulating the expression of VEGF and altering tumor proliferation characteristics and vascular distribution patterns, making it a promising novel therapeutic agent for OSA-associated lung cancer.

Despite the potential adjuvant therapeutic value of COX inhibitors, key research gaps remain: subtype-specific clinical studies are needed to clarify their differential effects on proliferation and metastasis of lung cancer with different driver gene mutations, while balancing their vascular regulatory effects with risks such as gastrointestinal side effects and altered CBF. Future research should focus on combined therapeutic regimens of COX inhibitors with CPAP and targeted drugs, exploring their synergistic effects in improving the tumor microenvironment and reducing oxidative stress burden to provide new strategies for the precise treatment of OSA-associated lung cancer.

### 5.2 Anti-angiogenic therapies

CIH related to OSA is a core pathological factor driving tumor angiogenesis in lung cancer. By activating HIF-1/VEGF pathway, it promotes the release of angiogenic factors, disrupts the balance of tumor angiogenesis, and accelerates tumor progression (Sánchez-de-la-Torre et al., 2023; Marrone and Bonsignore, 2020). This process involves the abnormal regulation of multiple key molecular pathways and biomarkers, providing clear targets for anti-angiogenic therapy, as detailed below.

#### 5.2.1 Endothelin axis-targeted therapy

Endothelin-1 and its receptors play a crucial role in CIH-induced cancer progression, exacerbating the malignant phenotype by promoting tumor cell proliferation, migration, angiogenesis, metastasis, and chemoresistance. A recent study confirmed that therapeutic blocking of endothelin receptors can effectively prevent CIH-induced tumor formation in both *in vitro* and *in vivo* models (Minoves et al., 2022), providing direct experimental evidence for anti-angiogenic therapy of OSA-associated lung cancer and promising to be one of the precise targeted strategies.

#### 5.2.2 VCAM-1-targeted therapy

Vascular cell adhesion molecule-1 (VCAM-1) is a key biomarker for vascular microenvironment remodeling in OSA-associated lung cancer. A clinical cohort study showed that circulating VCAM-1 levels in OSA patients are significantly associated with cancer risk (Hirsch Allen et al., 2024). It promotes tumor invasion and metastasis by mediating the adhesion and transendothelial migration of tumor cells and endothelial cells (Schlesinger and Bendas, 2015; Dymicka-Piekarska et al., 2012); moreover, VCAM-1 levels in patients with OSA combined with melanoma are significantly higher than those in tumor patients without OSA (Dymicka-Piekarska et al., 2012), indicating its specific role in vascular infiltration of OSA-related tumors. Therefore, the development of VCAM-1-specific inhibitors or antibody drugs can inhibit tumor vascular infiltration and distant metastasis by blocking the interaction between tumor cells and vascular endothelium, which is particularly suitable for OSA-associated lung cancer patients.

In summary, anti-angiogenic therapy, by targeting the abnormal angiogenesis pathway mediated by OSA-related CIH, holds promise as an effective adjuvant treatment for OSA-associated lung cancer. This therapeutic strategy is particularly suitable for combination with CPAP, chemotherapy, or targeted therapy to further improve efficacy. However, the safety and effectiveness of relevant strategies still need to be verified by large-scale clinical trials, and the suitable populations among different lung cancer subtypes need to be clarified.

#### 5.2.3 Endostatin-based combined therapy

Endostatin is an endogenous anti-angiogenic glycoprotein hydrolyzed from the carboxyl terminal of extracellular matrix collagen (Poluzzi et al., 2016). It can achieve tumor microenvironment inhibition and vascular normalization recovery by inhibiting VEGF and its signaling pathway, and regulating factors such as HIF-1α, MMPs, and basic fibroblast growth factor (bFGF) (Limaverde-Sousa et al., 2014; Walia et al., 2015; Fukumoto et al., 2005; Li K. et al., 2018). Clinical studies have shown that circulating endostatin levels are elevated in OSA patients (HR = 1.45, 95% CI = 1.12–1.87, P = 0.005) (Hirsch Allen et al., 2024), which is speculated to be a compensatory response to the pro-angiogenic microenvironment induced by OSA (Ärnlöv et al., 2013), but a single elevation is insufficient to inhibit tumor progression. In the CIH-induced OSA-associated lung cancer mouse model, endostatin can reduce cell proliferation and angiogenesis by inhibiting VEGF expression (Zhang et al., 2019), suggesting its potential therapeutic value. Given the complexity of angiogenesis imbalance in OSA patients, endostatin needs to be used in combination with other anti-angiogenic drugs such as VEGF inhibitors to enhance the blocking effect on abnormal angiogenesis and improve therapeutic efficacy.

### 5.3 Antioxidants

CIH related to OSA activates the HIF-1α and NF-κB pathways. This not only significantly promotes reactive oxygen species (ROS) production but also impairs the function of endogenous antioxidant defense systems, including superoxide dismutase (SOD), glutathione (GSH), and catalase (CAT) (Dharshini et al., 2023; Tian et al., 2022; Ntalapascha et al., 2013; Mancuso et al., 2012; Yu et al., 2019). Ultimately, these changes lead to DNA damage, genomic instability, and deterioration of the TME, accelerating lung cancer progression. Given this background, antioxidants represent a potential adjuvant treatment strategy for OSA-associated lung cancer by neutralizing ROS and repairing oxidative damage.

As a classic non-enzymatic antioxidant, vitamin E can specifically alleviate lipid peroxidation damage in OSA patients. Clinical studies have confirmed that vitamin E levels are significantly reduced in OSA patients (p < 0.006) (Sales et al., 2013). Exogenous supplementation can improve the oxidative stress microenvironment of OSA-associated lung cancer by directly neutralizing ROS and inhibiting lipid peroxidation, thereby creating an unfavorable condition for tumor cell proliferation. Tempol, a redox-cycling nitroxide radical, possesses unique dual antioxidant advantages: it can efficiently scavenge ROS (especially superoxide anions) and enhance endogenous SOD activity. Its neuroprotective effects have been verified by inhibiting peroxynitrite-related inflammatory responses (Lipman et al., 2006; Amankwa et al., 2023). Previous studies have shown that Tempol exerts protective effects against neurological diseases, cardiovascular diseases, and cancer, with core mechanisms closely related to reducing oxidative stress and inhibiting inflammatory pathways (Zhao et al., 2018; Rossetto et al., 2023; Park, 2022). More importantly, Tempol can directly inhibit HIF-1α-driven tumor progression (Ma et al., 2013; Nazarewicz et al., 2013), and HIF-1α is the key molecular target for CIH-induced lung cancer progression. In a CIH-induced OSA-like lung metastasis mouse model, Li et al. further confirmed that Tempol could significantly reduce CIH-induced oxidative stress, inflammatory responses, and melanoma lung metastasis (Li L. et al., 2018), providing direct experimental evidence for its application in OSA-associated lung cancer.

Although basic experiments and mechanistic studies have provided solid theoretical support (Lavalle et al., 2024), suggesting that antioxidants may benefit OSA-associated lung cancer patients unable to tolerate high-intensity treatment, specialized clinical studies are still scarce. Tempol is currently in the clinical trial stage, and most studies are based on animal models. Its efficacy, safe dosage, and long-term side effects in humans have not been clarified, requiring further large-scale prospective clinical trials for verification. Future efforts should combine subtype-specific research and combined therapy exploration to promote the clinical translation of antioxidant intervention for OSA-associated lung cancer.

## 6 Conclusions and future directions

### 6.1 Conclusions

**FIGURE 2:**
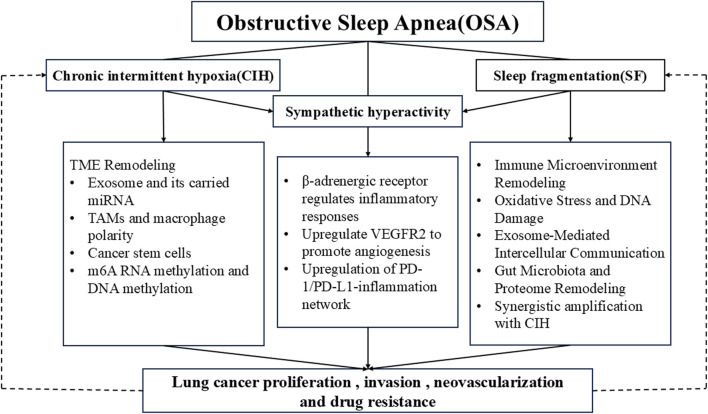
Flowchart illustrating the bidirectional interaction between OSA and lung cancer progression. It highlights how OSA’s core pathophysiological features—chronic intermittent hypoxia (CIH), sleep fragmentation (SF), and sympathetic hyperactivity—synergistically drive tumor microenvironment remodeling, oxidative stress, immune suppression, and angiogenesis, ultimately promoting lung cancer proliferation, invasion, neovascularization, and drug resistance. Conversely, lung cancer and its treatment can exacerbate CIH and SF, forming a bidirectional pathological cycle. TME, tumor microenvironment; TAMs, tumor-associated macrophages; VEGFR2, vascular endothelial growth factor receptor 2.

This narrative review synthesizes relevant cellular and molecular studies to delineate the potential pathophysiological mechanisms underlying the bidirectional crosstalk between OSA and lung cancer (Figure 2). In recent years, the TME has drawn much attention as a potential target for cancer immunotherapy (Schulz et al., 2019; Keremitçi et al., 2025). Mechanistically speaking, OSA-associated hypoxia modulates the TME via multiple synergistic pathways, with oxidative stress and chronic inflammation induced by CIH acting as core regulatory hubs. Specifically, on the one hand, CIH promotes exosome secretion by tumor cells; these exosomes induce M2 polarization of tumor-associated macrophages via their carried miR-106a-5p (Ren et al., 2024) and simultaneously upregulate PD-L1 expression on macrophage surfaces (Liu Y. et al., 2022), ultimately establishing an immunosuppressive microenvironment that impairs anti-tumor immunity. On the other hand, CIH activates the HIF-1α/ATAD2 and HIF-1α/ESM1 pathways to upregulate the expression of cancer stem cell markers such as CD133 and CD44, enhancing the invasive and metastatic capabilities of lung cancer cells (Gu et al., 2021; Hao et al., 2022). Notably, SF also participates in regulating this process via CD8^+^ T cells (Akbarpour et al., 2017). Meanwhile, sympathetic hyperactivity accelerates tumor progression and increases metastasis incidence through the βARs signaling pathway, while SF further promotes tumor growth and angiogenesis via the same pathway (Powell et al., 2013; Cui et al., 2021; Sun et al., 2024; Wheeler et al., 2021; Lourenço et al., 2022). Collectively, these findings indicate that CIH, SF, and sympathetic hyperactivity synergistically drive TME remodeling, thereby facilitating lung cancer initiation and progression.

A vicious cycle defines the bidirectional interplay between OSA and lung cancer: airway obstruction and inflammatory responses caused by lung cancer itself, along with tumor hypoxia and sleep disturbances induced by anti-tumor therapies (chemotherapy, radiotherapy, targeted therapy), may trigger or worsen OSA; conversely, OSA accelerates tumor progression via the aforementioned pathways. These regulatory pathways do not operate in isolation but are interconnected through oxidative stress and inflammatory factors (e.g., TNF-α, IL-6, IL-8) as intermediate mediators, ultimately forming a pro-tumorigenic network of “CIH/SF/sympathetic hyperactivity - oxidative stress/chronic inflammation - immunosuppression/immune escape.”

Nevertheless, several gaps remain in current research: first, a few studies have not found a significant association between OSA and lung cancer incidence or progression (Gozal et al., 2016; Brenner et al., 2019), which may be related to differences in study design, small sample sizes, or short follow-up periods; second, published Mendelian randomization (MR) analyses contradict the initial hypothesis, failing to provide conclusive evidence for a causal relationship and merely offering directions for mechanistic exploration (Yao et al., 2024), indicating that core drivers (e.g., common confounders, mediating pathways) of their association require further investigation—this gap may hinder comprehensive clinical management of both conditions, impeding high-risk population identification and intervention optimization; third, research into the reverse effect of lung cancer itself and its treatment on OSA is even scarcer. Lung cancer research has long focused on diagnosis and treatment, with insufficient attention to bidirectional crosstalk (Yuan et al., 2024). While this review briefly outlines potential mechanisms of lung cancer-induced OSA across three dimensions (tumor itself, oxidative stress, systemic inflammation, and anti-tumor therapy side effects), most conclusions rely on reasonable inferences from existing mechanisms, necessitating targeted studies to clarify specific molecular mechanisms and clinical features. Fourth, lung cancer is highly heterogeneous. This review only addresses associations between OSA and common histological subtypes (e.g., adenocarcinoma, squamous cell carcinoma, small cell carcinoma) as well as genotype-specific lung cancer (e.g., EGFR, KRAS, ROS1). Due to the extreme paucity of studies on OSA and lung cancer with unique molecular profiles, current evidence cannot support refined subtype analysis, requiring targeted experimental and clinical research to dissect subtype-specific associations and mechanisms.

In terms of clinical translation, research on combined therapy for OSA-associated lung cancer is extremely limited. Existing explorations are mostly in the preclinical stage, using animals or cells as experimental subjects with inherent species differences; moreover, the modeling duration is inconsistent with the natural course of OSA in humans, making it difficult to simulate the complex human microenvironment. The efficacy and potential side effects of related drugs remain unclear, and there is a long way to go before such drugs are officially applied in clinical application. Nevertheless, screening and targeted intervention for OSA patients with concurrent lung cancer still hold significant clinical value. As the most commonly used basic treatment for OSA patients, CPAP has been preliminarily shown to potentially improve the prognosis of OSA-associated lung cancer patients (Khalyfa et al., 2022; Srivali and De, 2025) (CPAP is not elaborated in detail in this review). Future randomized controlled trials (RCTs) are urgently needed to verify the efficacy of CPAP and other interventions, providing high-quality evidence for clinical decision-making. In summary, OSA can be regarded as a modifiable target for lung cancer prevention and treatment. Future targeted drugs developed against the aforementioned molecular pathways are expected to reverse OSA-related tumor therapy resistance and improve patient survival and prognosis.

### 6.2 Limitations

As a narrative review, this work has inherent limitations. Specifically, these limitations include two aspects: first, the selection of literature and extraction of data rely on the subjective judgment of researchers, which may introduce selection bias; second, the lack of quantitative synthesis methods, such as meta-analysis, prevents the quantification of the strength of the association between OSA and lung cancer. Despite these limitations, this review systematically summarizes the potential mechanisms by which OSA affects lung cancer progression through CIH, SF, and sympathetic hyperactivity, regulating tumor microenvironment components, including exosomes, TAMs, CSCs, and epigenetic modifications. It thus provides a clear theoretical framework for future research on the association between the two conditions.

### 6.3 Priority and directions of future research

Existing evidence supports a plausible association between OSA and lung cancer, but this association is regulated by multiple factors, and the strength of evidence is limited. Future research should be carried out in order of priority. First, conduct large-scale, long-term follow-up prospective cohort studies to clarify the causal relationship between OSA and lung cancer, focusing on the impact of regulatory factors such as hypoxia severity, gender, and age. Second, integrate and analyze the molecular pathways mediating exosome secretion, as well as their cross-regulatory mechanisms with OSA’s core pathological features and intercellular communication in the tumor microenvironment, while verifying the impact of CPAP treatment on the prognosis of patients with comorbidities. Third, strengthen research on the reverse effect of lung cancer itself and its treatment on OSA to fill existing research gaps. Finally, further explore the association between lung cancer of different subtypes and molecular characteristics and OSA to provide a basis for personalized treatment. In the long run, relying on big data platforms and gene databases, integrating multi-omics and radiogenomics results, and using machine learning technology for in-depth data mining can systematically reveal the intrinsic association and interaction mode between OSA and lung cancer, ultimately providing reliable theoretical support for formulating optimal clinical management strategies for OSA-associated lung cancer.
